# Lipase-Catalyzed Chemoselective Ester Hydrolysis of Biomimetically Coupled Aryls for the Synthesis of Unsymmetric Biphenyl Esters

**DOI:** 10.3390/molecules24234272

**Published:** 2019-11-23

**Authors:** Janna Ehlert, Jenny Kronemann, Nadine Zumbrägel, Matthias Preller

**Affiliations:** 1 Institute for Biophysical Chemistry, Hannover Medical School, 30625 Hannover, Germany; 2 Centre of Biomolecular Drug Research (BMWZ), Leibniz University Hannover, 30167 Hannover, Germany

**Keywords:** lipases, biphenyl esters, biocatalysis, chemoselectivity, biomimetic synthesis, organic synthesis

## Abstract

Lipases are among the most frequently used biocatalysts in organic synthesis, allowing numerous environmentally friendly and inexpensive chemical transformations. Here, we present a biomimetic strategy based on iron(III)-catalyzed oxidative coupling and selective ester monohydrolysis using lipases for the synthesis of unsymmetric biphenyl-based esters under mild conditions. The diverse class of biphenyl esters is of pharmaceutical and technical relevance. We explored the potency of a series of nine different lipases of bacterial, fungal, and mammalian origin on their catalytic activities to cleave biphenyl esters, and optimized the reaction conditions, in terms of reaction time, temperature, pH, organic solvent, and water–organic solvent ratios, to improve the chemoselectivity, and hence control the ratio of unsymmetric versus symmetric products. Elevated temperature and increased DMSO content led to an almost exclusive monohydrolysis by the four lipases *Candida rugosa* lipase (CRL), *Mucor miehei* lipase (MML), *Rhizopus niveus* lipase (RNL), and *Pseudomonas fluorescens* lipase (PFL). The study was complemented by in silico binding predictions to rationalize the observed differences in efficacies of the lipases to convert biphenyl esters. The optimized reaction conditions were transferred to the preparative scale with high yields, underlining the potential of the presented biomimetic approach as an alternative strategy to the commonly used transition metal-based strategies for the synthesis of diverse biphenyl esters.

## 1. Introduction

Selective transformation of one specific functional group in a molecule, containing multiple groups of similar reactivity is of the utmost relevance for targeted synthesis of molecules with pharmaceutical activities, as well as materials with well-defined properties for technical applications. Particularly, ester hydrolysis is one of the most fundamental chemical transformations. Successful strategies for the preferential monohydrolysis of a number of different esters have been reported under often strong basic conditions [1,2,3], however general applicability is limited by competing elimination reactions under these conditions and chemoselectivity regarding a wide spectrum of multiester compounds. No study has yet been reported for the selective transformation of biphenyl-based esters using lipase biocatalysis. Biphenyl-based building blocks with ester functions represent an important class of precursors that give rise to compounds with anti-infective, anti-inflammatory, and anti-cancer potencies [4,5,6,7,8,9,10], as well as in technical applications, such as in solar cells, where they are used as linkers for dyes [11] and hole transport materials [12], in metal organic frameworks for gas storage [13], and as catalysts [13,14] in lithium ion batteries [15] or rectification devices [16], in nanocomposite thin films for use in organic light emitting diodes (OLED) [17], or even as material in HPLC columns for the isolation of aromatic compounds [18]. Chemoselective hydrolysis and subsequent functionalization of biphenyl ester-based compounds therefore allow the generation of tailored molecules with increased potency and selectivity for their biological targets, improved bioavailability (absorption-distribution-metabolism-excretion-toxicity, ADMET) profiles, or unique magnetic or electronic properties.

Biocatalysis strategies using lipases have become an attractive tool for modern organic synthesis in industry and academic research, due to the broad accepted substrate spectra and the usually mild conditions that can be applied, such as low temperatures and neutral pH values [19,20,21]. Their synthetic use reduces the risk of side reactions considering the high chemo-, regio-, and stereoselectivity of lipases [22,23]. Given their tolerance for organic solvents, varying the ratios of water and organic solvent mixtures provides a means to control enzymatic reaction and selectivities. In addition, it offers the advantage to even convert small molecules with low solubilities in aqueous medium, in order to biocatalytically obtain the desired target products. Lipases catalyze a plethora of different reactions, including hydrolysis, esterification, transesterification, and alcoholysis [24], the major physiological function of lipases however is the hydrolytic cleavage of triacylglycerides for energy production, and their ubiquitous expression and functions in diverse cellular compartments, which facilitate enzyme activity under a variety of conditions [19,20,21]. Industrial applications of lipases include the generation of important pharmaceuticals through transesterifications and ester cleavage [19,25,26,27]. They are additionally used for biodiesel production in food and flavor industry as well as waste water treatment and generation of agrochemicals [24]. In organic synthesis, lipases are often used for desymmetrization and dynamic kinetic resolution towards enantiopure compounds [28,29]. Furthermore, lipases have been shown to biocatalytically convert benzylic esters [30,31]. However, the conversion of symmetric biphenyl-based ester building blocks to unsymmetric esters has not yet been investigated. 

Here, we present the combination of eco- and cost-friendly biomimetic oxidative phenol coupling with lipase-catalyzed monohydrolysis of biphenyl esters for the targeted synthesis of unsymmetric biphenyl-based ester building blocks. We systematically analyzed the catalytic activities of a series of lipases from different organisms for their potency to hydrolyze biphenyl esters. We are investigating particularly the effect of the reaction conditions, in terms of water–organic solvent ratios, pH, type of solvents, and reaction time, on the efficacy and chemoselectivity of the lipases, as well as their accepted substrate spectrum. Accompanying molecular docking experiments helped to rationalize the observed catalytic activities and chemoselectivities. Our study provides a biomimetic strategy using lipase catalysis for the controlled synthesis of unsymmetric biphenyl ester building blocks under mild conditions. 

## 2. Results

### 2.1. Biomimetic Synthesis of Biphenyl Ester Building Blocks

**Scheme 1:**
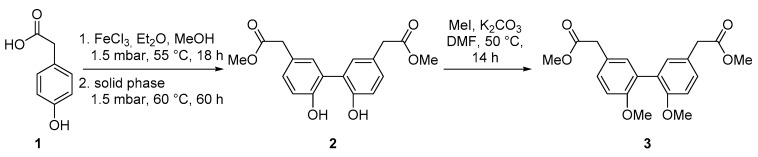
Biomimetic iron(III)-catalyzed synthesis route towards symmetric biphenyl esters **2** and **3**.

Biphenyl esters have been reported as important building blocks for the development of pharmaceuticals in diverse indication areas, and for use in a wide range of technical applications [4,5,6,7,8,9,10,11,12,13,14,15,16,17,18]. In order to allow an inexpensive, environmentally friendly, and efficient access to biphenyl ester core moieties, we adopted a biomimetic iron(III)-catalyzed, solid-phase coupling reaction. In nature, this type of chemical reaction is usually used by plants to metalloenzymatically produce specific metabolites required for plant growth and protection [32,33,34]. Fe(III)-catalyzed oxidative coupling of aryls and phenols have been reported for the synthesis of natural products under mild conditions [35,36]. We obtained C–C bond formation through oxidative coupling of commercially available 4-hydroxyphenyl acetate (**1**) using iron(III) chloride on silica with 60% yield of biphenyl ester **2** (Scheme 1). This reaction builds mechanistically on mesomeric effects due to the ortho substitution with the electron donating hydroxyl group. Iron(III) chloride acts as the catalyst and is reduced to allow oxidative phenol coupling (Scheme S1). In a second step, the purified biphenyl ester **2** was converted to biphenyl ester **3** through methylation of the free hydroxyl groups at the biphenyl scaffold using methyl iodide with 90% yield. 

**Scheme 2:**
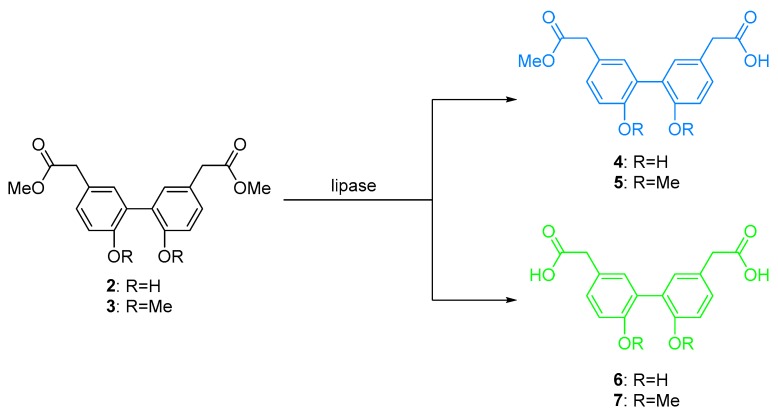
Schematic representation of lipase catalyzed ester hydrolysis of biphenyl esters. Depending on the chemoselectivity of the lipases, biphenyl esters **2** and **3** were converted to either unsymmetric acids **4** and **5** or symmetric acids **6** and **7**, respectively.

Subsequently, the biphenyl ester building blocks **2** and **3** were subjected to a lipase-catalyzed ester hydrolysis to yield either the unsymmetric acids **4** and **5,** or the symmetric acids **6** and **7**, respectively (Scheme 2). To gain control over the obtained products, we tested a series of lipases, and systematically varied the reaction conditions and reaction time. As a control, we synthesized biphenyl ester building blocks without substituents at the ortho position of the biphenyl moiety, in order to examine the accessible substrate spectrum of the lipases for differently substituted biphenyl ester building blocks.

### 2.2. Chemical Synthesis of Biphenyl Ester Building Blocks without Ortho Substituents

**Scheme 3:**
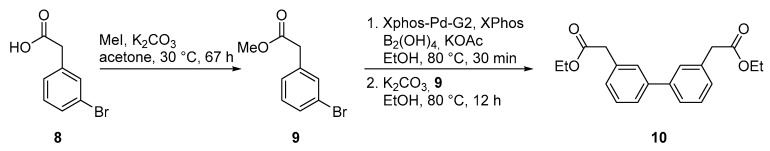
Synthesis of the biphenyl ester building block without ortho substitutions at the core moiety (**10**) through methylation of 3-bromo phenyl acetic acid (**8**), followed by Suzuki–Miyaura cross coupling.

To examine the substrate spectrum and the impact of the ortho substitution at the biphenyl core moiety on the hydrolysis by lipases, we synthesized biphenyl esters without ortho substituents at the core moiety. Since in the absence of ortho substitutions the iron(III)-catalyzed oxidative phenol coupling is not applicable for the synthesis of biphenyl ester **10**, we used a palladium-catalyzed Suzuki–Miyaura cross-coupling reaction of the aryl bromide (**9**) and an in situ generated phenyl boronic acid using the XPhos-Pd-G2 catalyst and XPhos as the ligand (Scheme 3). Methyl 2-(3-bromophenyl)acetate (**9**) was synthesized from the commercially available 3-bromo phenyl acetic acid (**8**) by methylation using methyl iodide with quantitative yields. 

**Scheme 4:**
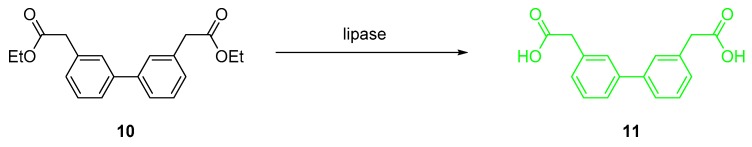
Biocatalytic ester cleavage of the symmetric compound **10** using lipases gave exclusively the symmetric acid **11.** The unsymmetric product was intermediately detected only in traces.

Lipase-catalyzed ester hydrolysis of the symmetric biphenyl ester **10** resulted exclusively in ester cleavage of all available ester groups in the molecule and the formation of the symmetric biphenyl-based carboxylic acid **11** (Scheme 4). 

### 2.3. Activity and Chemoselectivity of Lipases on Biphenyl Ester Hydrolysis

In our attempt to optimize the biocatalytic ester hydrolysis of biphenyl-based esters, we initially tested the hydrolysis activity of a series of nine lipases from different organisms (Table 1) on the conversion of biphenyl esters **2** and **3** using a colorimetric assay. Bromothymol blue (BTB) served as the pH indicator to determine the formation of free carboxylic acids, indicated by a color change from blue to yellow (Figure S1). The enzymatic reactions were performed in 96-well format in phosphate buffer, pH 7.4, and 10% DMSO (*v*/*v*) at room temperature. As a positive control, glyceryl tributyrate (TAG (**13**)) was hydrolyzed by all lipases. At the given conditions, *Candida antarctica* Lipase B (CALB) was found to be able to cleave the biphenyl esters **2** and **3** to release the corresponding carboxylic acids (Table 1). Exchanging the organic solvent DMSO with either water-insoluble toluene or water-soluble acetonitrile, we did not detect any acid formation even for CALB (Figure S1).

**Figure 1:**
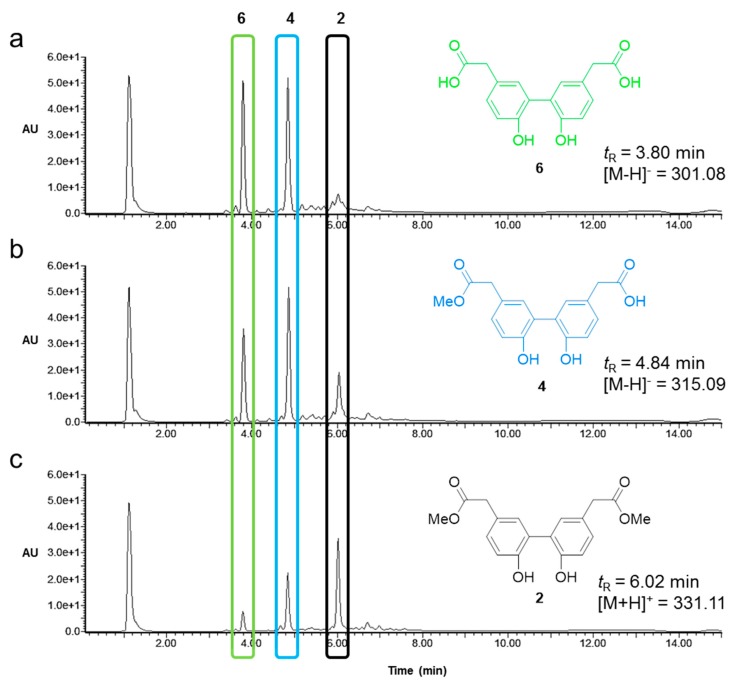
Time dependency of the chemoselective hydrolysis of biphenyl ester **2** with free hydroxyl groups at the core moiety obtained by analytical LC-MS and comparison of the UV-signal ratios of biphenyl esters **2** (black), **4** (blue), and **6** (green). All reactions were performed at 25 °C, pH 8.0, and 10% DMSO (*v*/*v*) using CALB. (**a**) Reaction time: 3 h; (**b**) reaction time: 2 h; (**c**) reaction time: 1 h. The chemical structures, retention times, and *m*/*z* values for compounds **2**, **4**, and **6** are given.

To assess the impact of the reaction time on the enzymatic ester hydrolysis, we monitored the formation of the hydrolysis products during lipase catalysis via analytical LC-MS. We found that hydrolysis of biphenyl ester **2**, containing free hydroxyl groups at the core moiety by CALB, gave a mixture of unsymmetric and symmetric acids **4** and **6** already after 1 h. The ratio was dependent on the reaction time and the pH value (Figure 1, Figure S2). Longer reaction times led to a shift of the product ratio towards the fully hydrolyzed, symmetric acid **6** (Figure 1). The most chemoselective conversion with CALB towards the unsymmetric acid **4** was determined for a reaction time of 2 h at pH 8.0, giving a product ratio of 20%:50%:30% (**2**:**4**:**6**). In order to analyze the catalytic conversion of other biphenyl ester building blocks and examine the substrate spectrum of the lipase regarding ortho substitutions at the biphenyl core moiety, we performed the same analytical LC-MS analysis using biphenyl esters **3** and **10**. In both cases, biocatalytic hydrolysis under the same conditions using CALB led to a favorable hydrolytic ester cleavage to the symmetric acids **7** (50–75%) and **14** (90%) at all examined pH values (Figures S3 and S4). However, at a reaction time of 2 h, the unsymmetric product **5** with methoxy groups at the ortho position of the biphenyl rings was clearly detectable (13%,) while the major product was still the symmetric acid **6** (42%) (Figure S3). Therefore, CALB shows sufficient promiscuity to accept a series of ortho substituted biphenyl esters as substrates with time and pH dependencies regarding the chemoselective hydrolysis of the two ester groups.

**Figure 2:**
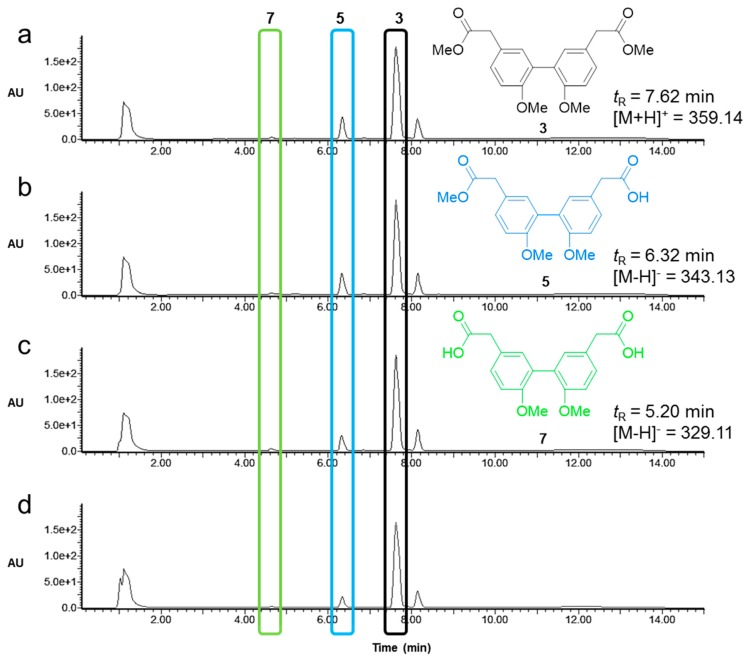
Chemoselective hydrolysis of the biphenyl ester **3** with methoxy groups at the core moiety to the unsymmetric product **5** by four lipases at increased DMSO concentration, temperature, and reaction time, obtained by analytical LC-MS from the UV-signal ratios of compounds **3** (black), **5** (blue), and **7** (green). The reaction time for all experiments was 48 h at 37 °C, pH 8.0, and 40% DMSO (*v*/*v*). Chemical structures, retention times, and *m*/*z* values of 3, 5, and 7 are given. (**a**) Biocatalysis by PFL; (**b**) biocatalysis by MML; (**c**) biocatalysis by RNL; (**d**) biocatalysis by CRL.

Motivated by the result that the unsymmetric acid **5** with methoxy groups was also detectable, and previous studies, which reported a maximal catalytic activity of the applied lipases at elevated temperature and organic solvent content [37], we varied systematically the temperature and reaction time of the lipase-catalyzed hydrolysis of biphenyl ester **3** at an increased organic solvent content of 40% DMSO (*v*/*v*) (Table S1). Unfortunately, CALB rapidly led to the unwanted symmetric product **6** as shown before. The four lipases *Rhizopus niveus* lipase (RNL), *Mucor miehei* lipase (MML), *Candida rugosa* lipase (CRL), and *Pseudomonas fluorescens* lipase (PFL) were found to be active to hydrolyze compound **3** in the colorimetric lipase activity assay in the presence of the elevated DMSO concentration. Using analytical LC-MS, we found a critical dependency of the increased DMSO content, reaction time, and temperature on the chemoselective conversion of compound **3** to the unsymmetric product **5**. Increasing the DMSO concentration to 40% (*v*/*v*) led to a reduced reaction velocity of the lipase biocatalysis, indicated by the small amounts of formed acids even after 24 h at temperatures of both 25 and 30 °C (<5% of compound **5**). Elevated temperatures improved the catalytic activity of the four lipases, with reactant (**3**):unsymmetric product (**5**) ratios for the four lipases CRL, MML, RNL, and PFL of 6.7:1 (**5**: 13%), 3.6:1 (**5**: 22%), 6:1 (**5**: 14%), and 3.5:1 (**5**: 22%), respectively after 48 h at 37 °C. All four lipases exhibited a high chemoselectivity towards the formation of the unsymmetric acid **5**, without detectable cleavage of the second ester group (i.e., the formation of the symmetric acid **7** in the presence of 40% DMSO (*v*/*v*) after 48h at 37 °C and pH 8.0) (Figure 2). 

### 2.4. Molecular Docking Experiments

Although various studies showed the promiscuity of lipases to allow broad substrate spectra, our results clearly revealed that the catalytic activity and chemoselectivity of the studied lipases can be controlled and strictly depend on the reaction conditions, particularly the pH values, the amount of organic solvent, as well as the reaction time and temperature. The optimal reaction conditions differed slightly for the different lipases, but eventually led to highly selective monohydrolysis of compounds with several ester functions and the synthesis of unsymmetric biphenyl-based ester building blocks. 

**Figure 3:**
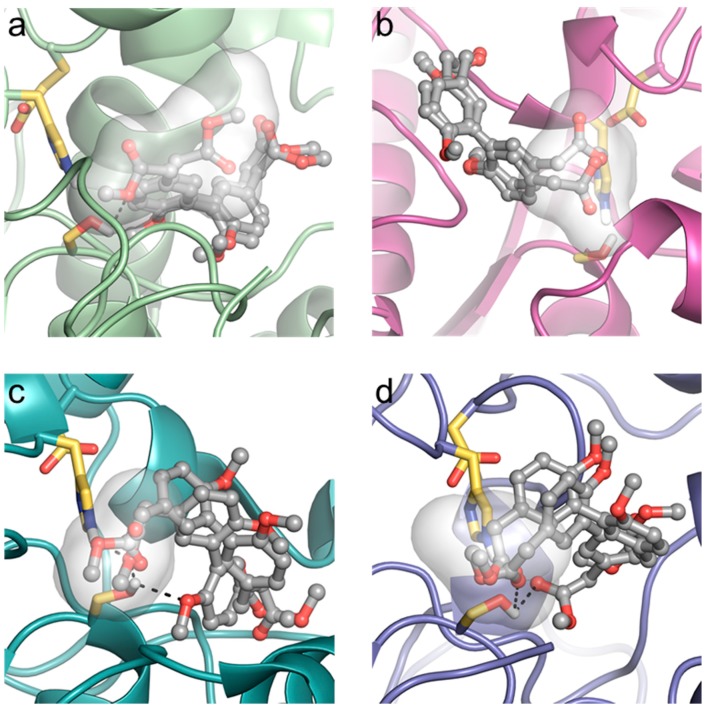
Predicted binding poses of biphenyl ester **3** (grey) to the active sites of the four lipases (**a**) CRL (green), (**b**) RNL (magenta), (**c**) MML (cyan), and (**d**) PFL (blue) using molecular docking. Two representative conformations of the largest cluster of docking solutions are shown. The serine, histidine, and glutamate/aspartate residues of the catalytic triad are show in sticks representation (yellow). Hydrogen-bonding interactions are shown as dotted lines. The variation in the orientation of the ester group in the active site is indicated by the transparent surface, as obtained from all conformations within the cluster.

These four identified lipases showed differing efficacies in converting the reactant **3** to the unsymmetric product **5** (Figure 2). To rationalize the different efficacies of the four lipases, we performed complementing in silico binding predictions of the biphenyl ester reactant **3** to the four lipases CRL, RNL, MML, and PFL using molecular docking. Molecular docking provides structural insights into the binding of the small molecule biphenyl esters to the lipase enzymes—in terms of their predicted binding poses and binding affinities—which is a prerequisite for the biocatalytic conversion of the compounds. The active site of lipases contains a highly conserved catalytic triad, consisting of a serine, a histidine, and a negatively charged glutamate or aspartate residue [38,39]. The biphenyl ester **3** was predicted to bind in the active site of all four lipases, in close proximity to the catalytic triad, however with slightly different binding poses and positions (Figure 3). The ester group of **3** in the active site shows larger variations in its orientation for the binding to CRL and RNL, while for the binding to MML and PFL, this ester group remains mostly bound to the catalytically active serine. In accordance with the different poses, the computed binding affinities to the four lipases varied between −3.23 and −6.79 kcal·mol^−1^ (Table 2). These predicted binding affinities correlate well with the observed biocatalytic efficacies of the lipases to convert reactant **3** to the unsymmetric biphenyl ester **5**. 

### 2.5. Lipase-Catalyzed Biphenyl Ester Conversion in Preparative Scale

**Scheme 5:**
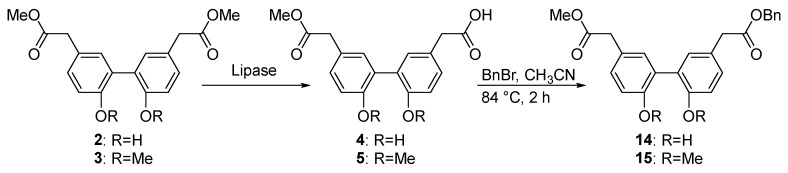
Two-step strategy for the lipase-catalyzed conversion of the symmetric ester reactants to unsymmetric biphenyl esters. Conditions for the biocatalytic conversion of **2**: CALB, 25 °C, 2 h. Conditions for the biocatalytic conversion of **3**: PFL, 37 °C, 48 h.

After successfully establishing the chemoselective hydrolysis of biphenyl esters using lipase biocatalysis, we demonstrated the enzyme-catalyzed reaction of **2** and **3** at a preparative scale to be able to isolate the unsymmetric acids **4** and **5** for subsequent benzylation of the free carboxylic acid with benzyl bromide to yield the unsymmetric biphenyl esters **14** and **15** containing both a methyl ester and a benzylic ester group (Scheme 5). 

We isolated the highly pure unsymmetric acid **5** with 41% yield. Synthesis of the unsymmetric biphenyl ester products **14** and **15** without purification of the intermediates gave yields of 20% and 12%, respectively. However, during the two-step synthesis of **14**, a byproduct (**16**) was formed (40%, Scheme S2), which showed benzylated phenolic hydroxyl groups. Unsatisfied by the moderate yields, we repeated the two-step reaction, while isolating the intermediate acid, and we obtained yields of 81% for the successful synthesis of unsymmetric biphenyl ester **15** (35% o2s). 

## 3. Discussion

Lipases are versatile biocatalysts for numerous targeted chemical transformations and widely used tools in organic synthesis, however their efficiency and selectivity to hydrolyze esters with biphenyl core moieties has not been examined so far. Here we established a biomimetic strategy for the chemoselective synthesis of unsymmetric biphenyl-based ester building blocks by combining iron(III)-catalyzed oxidative coupling reactions to construct the biphenyl core moiety, with selective monohydrolysis of the biphenyl diesters using lipases. This synthetic route offers a simple, environmentally friendly, and inexpensive access to unsymmetric biphenyl ester building blocks under mild conditions and can be used for the synthesis of a wide range of biphenyl esters with broad technical and pharmaceutical applications. We systematically addressed the impact of the reaction conditions on the catalytic activity and chemoselectivity of the lipases, particularly the effect of the reaction time and temperature, as well as the pH, water–organic solvent ratio, and type of organic solvent. 

The bioinspired oxidative phenol coupling using the cheap and non-toxic iron(III) catalyst FeCl_3_·6 H_2_O offered us an efficient solid-phase homocoupling of phenol esters in one step to produce symmetric biphenyl-based methylesters in high yields. In the second step, we used lipase biocatalysis to chemoselectively hydrolyze one of the two equivalent ester groups. For that purpose, we profiled a series of nine lipases from different organisms, covering bacteria, fungi, and vertebrates, for their efficacy to catalyze the hydrolytic ester cleavage of the biphenyl esters. Five of the lipases were found to be able to cleave the biphenyl esters under different reaction conditions with a marked dependency of the enzymatic activity and particularly the chemoselectivity on the reaction time, temperature, pH value, and organic solvent content. Our results are in good agreement with previous studies, which reported that the activity of some of the lipases was critically dependent on the temperature and DMSO concentration [37]. 

The identified active lipases CALB, CRL, RNL, MML, and PFL, capable of hydrolyzing biphenyl esters, showed a general acceptance of ortho substitutions at the biphenyl core moiety and hence, an advantageous level of promiscuity, allowing therefore their manifold use for biocatalytic conversions of a broad spectrum of biphenyl ester building blocks. For the biphenyl ester **2** with free hydroxyl groups in the ortho position of the core moiety, we found optimal reaction conditions that favor the formation of the unsymmetric product **4** by a factor of two over the symmetric product **6** using CALB at a moderate pH of 8, 25 °C, and 10% DMSO (*v*/*v*) after a reaction time of 2 h. Biphenyl **3** with methoxy groups at the biphenyl moiety was quickly converted to the symmetric product **7** under the same conditions. However, increasing the solvent content to 40% DMSO (*v*/*v*) and elevating the reaction temperature critically affected the activity and particularly the chemoselectivity of the four lipases CRL, MML, RNL, and PFL to efficiently and almost exclusively produce the unsymmetric biphenyl ester **5** by monohydrolysis of solely one ester group in the biphenyl diester. The chemoselective hydrolysis of only one ester group in the molecule was independent of the reaction time, and the overall yields increased steadily up to 48 h. The highest yields of unsymmetric biphenyl ester were obtained with lipases MML and PFL. The hydrolysis efficacies of the four lipases correlated well with predicted binding affinities by complementing computational docking experiments. Lipases such as PFL and MML featured favorable binding poses in proximity to the catalytic triad in the active site, and hence higher binding affinities of the biphenyl esters.

We were able to successfully transfer the optimized reaction conditions to a preparative scale and isolate the unsymmetric esters **4** and **5**. Interestingly, the yields of the purified unsymmetric products were significantly higher than the yields obtained at an analytical scale, underpinning the potential of using lipases as biocatalysts for the selective monohydrolysis of biphenyl esters. 

Biphenyl esters have been shown to represent an interesting class of building blocks for the development of pharmaceuticals and functionalized materials for use in a wide range of technical applications. This approach has great potential for the chemoselective conversion of a single functional group out of multiple groups with similar reactivities in a molecule, which can be challenging through traditional synthetic approaches, and the selective synthesis of unsymmetric biphenyls. Our biomimetic two-step strategy offers a series of advantages compared to other synthetic routes, such as the Suzuki–Miyaura coupling, the most frequently used synthetic approach for the synthesis of biphenyl core moieties, including no need for aryl halides, low cost, and mild reaction conditions, whereas the usually basic conditions of the Suzuki–Miyaura coupling include degradation of reactants, environmentally friendly, and no transesterifications by the solvent. In addition, the present study opens a novel facet for the effective and selective total synthesis of natural products, such as the pharmaceutically relevant ellagitannins, which possess biphenyl ester motifs connected to a glucopyranose, and which exhibit anti-infective, anti-inflammatory, and anti-cancer activities.

Summarizing our studies, we were able to establish a selective and efficient biomimetic strategy towards unsymmetric biphenyl esters by combining iron-catalyzed phenol coupling and lipase biocatalysis for the selective ester monohydrolysis under mild conditions. Varying critical reaction parameters, including time, pH, temperature, and solvent, control the activity and chemoselectivity of the enzymatic ester hydrolysis reaction. 

## 4. Materials and Methods 

### 4.1. Chemical Methods—General Information

Reactions with air- or moisture-sensitive reactants were performed under oxygen-free and dry inert atmosphere (nitrogen or argon). Chemicals were purchased from ABCR, Acros, AppliChem, AlfaAesar, Carl Roth, Fluka, Fluorochem, Sigma Aldrich, Merck and TCI and used without further purification. Dry solvents were freshly distilled or directly purchased from commercial suppliers and if not otherwise stated directly used without further purification. Silica gel column chromatography was performed under pressure with silica (60 Å, size 35–70 µM) purchased from Merck. TLC on silica gel was performed with silica coated glass slides from Merck (spot detection with UV or staining potassium-permanganate, ninhydrine or bromocresol green). Nuclear magnetic resonance spectra were recorded with a Bruker DPX-400 spectrometer. ^13^C NMR spectra were measured without ^1^H coupling. Chemical shifts were reported in ppm and calibrated to residual proton signal of deuterated solvents (CDCl_3_: *δ* = 7.26 ppm, CD_3_OD: *δ* = 3.31 ppm). Multiplicities are described using the following abbreviations: s = singlet, d = doublet, t = triplet, q = quartet, m = multiplet. ^13^C NMR spectra were reported as values relative to the residual solvent signal (CDCl_3_: *δ* = 77.16 ppm, CD_3_OD: *δ* = 49.00 ppm) as internal standards. High resolution mass spectra were obtained with Micromass LCT via loop-mode injection from a Waters (Alliance 2695) HPLC system with achieved ionization by electrospray ionization (ESI). Calculated and found masses are given in the synthetic procedures. 

### 4.2. Analytical LC-MS

Data from analytical LC–MS were obtained using a Waters 2767 sample manager connected to Waters 2545 pumps and system fluidics organizer (SFO), a Phenomenex Kinetex column (2.6 µ, C_18_, 100 Å, 4.6 × 100 mm) equipped with a Phenomenex Security Guard precolumn (Luna C_5_ 300 Å) eluted at 1.0 mL·min^−1^ with a Waters 1998 Diode Array detector (200–600 nm), Waters 2424 ELSD, and Waters SQD-2 mass detector operating simultaneously in ES^+^ and ES^-^ modes between 100 *m*/*z* and 650 *m*/*z*. The solvent gradient (**A**: HPLC grade *dd*H_2_O containing 0.05% formic acid; **B**: HPLC grade CH_3_CN containing 0.045% formic acid) was as follows: start: 10% **B**, 10 min 10%→90% **B**, 2 min 90% **B**, 1 min 90%→10% **B**, 2 min 10% **B** (total time: 15 min).

### 4.3. Preparative LC-MS

Purification of compounds with preparative LC-MS was done using a Waters mass-directed autopurification system comprising a Waters 2767 autosampler, a Waters 2545 pump system and SFO, and a Phenomenex Kinetex Axia column (5 µ, C_18_, 100 Å, 21.2 × 250 mm) equipped with a Phenomenex Security Guard precolumn (Luna C_5_ 300 Å), eluting at 20 mL·min^-1^ at ambient temperature (solvents as mentioned in analytical LC-MS). The post-column flow was split in a ratio 100:1 and the minority flow was made up of HPLC grade MeOH (0.045% formic acid) to 1 mL·min^−1^ for simultaneous analysis by diode array (Waters 2998), evaporative light scattering (Waters 2424), and ESI mass spectrometry in positive and negative modes (Waters SQD-2). Detected peaks were collected into glass test tubes. The solvent was evaporated, and the residue analyzed by NMR.

### 4.4. Dimethyl-2,2‘-(6,6‘-dihydroxy-[1,1‘-biphenyl]-3,3‘-diyl)diacetate (***2***)

Silica gel (3.62 g) was added to a solution of FeCl_3_ ∙ 6 H_2_O (1.76 g, 6.51 mmol) in diethyl ether (95 mL) and methanol (5 mL) at room temperature and stirred for 5 min. The reaction mixture was concentrated under reduced pressure and the obtained yellow solid was stirred for 18 h in vacuo (1.5 mbar) at 55 °C. A solution of 4-hydroxyphenyl acetic acid (**1**, 61.7 mg, 0.41 mmol) in dichloromethane (15 mL) was added at room temperature. After thorough mixing the solvent was evaporated. The remaining dark solid was stirred in vacuo (1.5 mbar) at 60 °C. After 60 h, the solid was dissolved in methanol (25 mL) and filtered through Celite. The solvent was removed in vacuo and the crude product was purified by silica gel chromatography (first column: petrol ether—EtOAc, 1:1; second column: petrol ether—EtOAc, 2:1) to provide a highly viscous, colorless oil of **2** (40.26 mg, 0.122 mmol, 60%): ^1^H-NMR (400 MHz, CDCl_3_): *δ* (ppm) = 7.17 (dd, *J* = 2.39 Hz, *J* = 8.19 Hz, 2H), 7.14 (d, *J* = 2.39 Hz, 2H), 6.91 (d, *J* = 8.19 Hz, 2H), 6.21 (bs, 2H), 3.70 (s, 6H), 3.59 (s, 4H). ^13^C-NMR (100 MHz, CDCl_3_): *δ* (ppm) = 172.73, 152.16, 132.18, 130.59, 126.71, 124.26, 117.09, 52.21, 40.18. HRMS *m*/*z* calculated for C_18_H_18_O_6_ Na [M + Na]^+^: 353.1003; found 353.1001.

### 4.5. Dimethyl-2,2′-(6,6′-dimethoxy-[1,1′-biphenyl]-3,3′-diyl)-diacetate (***3***)

Potassium carbonate (0.11 g, 0.78 mmol) was added to a solution of biphenyl acetate **2** (0.12 g, 0.36 mmol) in dry DMF (1 mL) at room temperature. The reaction mixture was heated to 50 °C and stirred for 45 min, before it was cooled to room temperature and a solution of methyl iodide (0.05 mL, 0.85 mmol) in dry DMF (0.1 mL) was added slowly. The mixture was stirred for 14 h at 50 °C, after which it was cooled to room temperature and quenched by the addition of H_2_O (13 mL). The organic layer was separated and extracted with CH_2_Cl_2_. NaOH (10%, 2.6 mL) was added to the combined organic layers and stirred for 2 h. The organic layer was separated, and the aqueous layer was extracted with CH_2_Cl_2_. The combined organic layers were washed with H_2_O, dried over Na_2_SO_4_, and concentrated. The crude product was purified by silica gel chromatography (petrol ether—EtOAc, 2:1) to yield a colorless oil of **3** (0.12 g, 90 %): ^1^H-NMR (400 MHz, CDCl_3_): *δ* (ppm) = 7.24 (dd, *J* = 2.05 Hz, *J* = 8.53 Hz, 2H), 7.15 (d, *J* = 2.05 Hz, 2H), 6.92 (d, *J* = 8.53 Hz, 2H), 3.76 (s, 6H), 3.69 (s, 6H), 3.59 (s, 4H). ^13^C-NMR (100 MHz, CDCl_3_): *δ* (ppm) = 172.37, 156.20, 132.36, 129.41, 127.57, 125.68, 111.19, 55.80, 52.01, 40.35. HRMS *m*/*z* calculated for C_20_H_22_O_6_ Na [M + Na]^+^: 381.1416; found 381.1314.

### 4.6. Methyl-2-(3-bromophenyl)acetate (***9***)

3-Bromo phenyl acetic acid (**8**, 4 g, 18.7 mmol, 1.0 eq) and dried K_2_CO_3_ (8.84 g, 56 mmol, 3.0 eq) were dissolved in acetone (40 mL) under inert conditions. Methyl iodide (10.5 mL, 56 mmol, 3.0 eq) was added dropwise and the reaction mixture was stirred for 67 h at 30 °C. After cooling the reaction to room temperature, the suspension was filtered with Celite and the solvent was removed in vacuo. The crude product was purified by silica gel chromatography (PE:EtOAc/4:1) yielding the product methyl-2-(3-bromophenyl)acetate (**9**) after evaporation as colorless oil in 100% yield (4.25 g, 18.7 mmol, quant.). R_f_ (PE:EtOAc/3:1): 0.85; ^1^H-NMR (400 MHz, CD_3_Cl): δ = 7.44 (d, *J* = 1.5 Hz, 1H), 7.41 (dt, *J* = 6.5, 2.2 Hz, 1H), 7.23–7.17 (m, 2H), 3.71 (s, 3H), 3.60 ppm (s, 2H); ^13^C-NMR (100 MHz, CD_3_Cl): δ (ppm) = 171.4, 136.1, 132.4, 130.3, 130.1, 128.0, 122.6, 52.2, 40.7. 

### 4.7. Diethyl 2,2′-([1,1′-biphenyl]-3,3′-diyl)diacetate (***10***)

The halogenated compound **9** (90 mg, 0.40 mmol, 1.0 eq), XPhos-Pd-G2 (3.1 mg, 0.004 mmol, 0.01 eq), XPhos (3.8 mg, 0.008 mmol, 0.02 eq), B_2_(OH)_4_ (106.2 mg, 1.20 mmol, 3.0 eq), and KOAc (116.3 mg, 1.20 mmol, 3.0 eq) were dissolved in ethanol under inert conditions. The reaction mixture was stirred for 30 min at 80 °C until the color changed to yellow, and then cooled down to room temperature. K_2_CO_3_ (272.9 mg, 2.0 mmol, 5.0 eq) and **9** (90 mg, 0.40 mmol, 1.0 eq) were added and the reaction mixture was stirred for 16 h at 80 °C. After cooling to room temperature, the reaction mixture was filtered with Celite and the solvent evaporated. The crude product was purified by silica gel chromatography (PE:EtOAc/9:1) yielding the biphenyl ester **10** as yellowish oil (40 mg, 0.014 mmol, 34%). R_f_ (PE:EtOAc): 0.64. ^1^H-NMR (400 MHz, CDCl_3_): δ (ppm) = 7.49 (d, *J* = 7.6 Hz, 4H), 7.39 (t, *J* = 7.8 Hz, 2H), 7.28 (d, *J* = 7.5 Hz, 2H), 4.17 (q, *J* = 7.1 Hz, 4H), 3.68 (s, 4H), 1.27 (t, *J* = 7.1 Hz, 6H). ^13^C-NMR (100 MHz, CDCl_3_): δ (ppm) = 171.5, 141.2, 134.6, 128.9, 128.2, 128.2, 1256.0, 60.9, 41.5, 14.2. HRMS (ESI) *m*/*z* calculated for C_20_H_22_O_4_ [M + H]^+^ = 327.1591, found: 327.1619.

### 4.8. Colorimetric Lipase Activity Screening

Lipases were purchased from Sigma Aldrich (Lipase basic kit). In every well of a 96-well microtiter plate protein (final concentration: 10 U· mL^-1^) was 180 µL phosphate buffer (10 mM, pH 7.4) with 0.01% bromothymol blue (BTB) mixed with 20 µL compound (100 mM in DMSO, CH_3_CN or toluene). The final concentration of the organic solvent in each well was 10% (*v*/*v*). For every lipase a negative control, containing only protein and BTB in phosphate buffer, as well as a positive control using glyceryl tributyrate (TAG (**13**), 100 mM in DMSO) were prepared. The plate was shaken gently for 28 h at room temperature and the color was monitored at 0, 1, 2, 4, 24, and 28 h, respectively. The assay was performed in duplicates. With lipases RNL, MML, PFL, and CRL, the assay was additionally performed in the presence of 40% DMSO (*v*/*v*).

### 4.9. Analysis of Lipase Activity and Selectivity by Analytical LC-MS

In total, 1 mg lipase in 180 µL phosphate buffer (50 mM, pH 7.4/8.0/9.0 (AMP-buffer)) was mixed with 20 µL compound (100 mM in DMSO) for all experiments with 10% DMSO (*v*/*v*). For experiments in the presence of 40% DMSO (*v*/*v*), 80 µL compound (25 mM in DMSO) was used. The reaction mixtures were shaken at 25 or 37 °C, and 20 µL samples were taken at the indicated time points for analytical LC-MS measurements. The reaction in the samples was stopped by the addition of 5 µL 2M HCl and diluted with 20 µL CH_3_CN. Analytical LC-MS measurements were performed as mentioned before. The product–reactant ratios were determined by comparing the UV signals from a diode array, where the ratio of peak areas was proportional to the ratio of concentrations.

### 4.10. Benzyl 2-(2′,6-dihydroxy-5′-(2-methoxy-2-oxoethyl)-[1,1′-biphenyl]-3-yl)acetate (***14***) and 2-(2′,6-Bis(benzyloxy)-5′-(2-methoxy-2-oxoethyl)-[1,1′-biphenyl]-3-yl)acetic acid (***16***) 

10.3 mg of CALB were dissolved in phosphate buffer (4 mL, 50 mM, pH 8.0) and biphenyl ester **2** (15 mg, 0.045 mmol, 1.0 eq) in DMSO (450 µL) was added to the mixture. The solution was stirred for 2 h at room temperature. To terminate the reaction 1 M HCl was added to adjust the pH to 1, and the aqueous layer was washed three times with EtOAc. The solvent was evaporated, and the residue was directly used for benzylation without any purification steps. The crude product was dissolved in CH_3_CN (10 mL), and 1,8-diazabicyclo(5.4.0)undec-7-ene (DBU) (1.5 mg, 0.01 mmol, 0.22 eq) and benzyl bromide (90 µL, 0.757 mmol, 16.8 eq) were added. The reaction mixture was stirred for 2 h at 84 °C. After cooling down *d*H_2_O was added and the aqueous layer was washed three times with EtOAc. The combined organic layer was dried over Na_2_SO_4_ and the solvent was evaporated. Purification of the crude product was performed using a Büchi Reverlis Prep equipped with a 4 g silica column (Ecoflex) and PE/EtOAc gradient; **14** eluted at 5% EtOAc and **16** at 50% EtOAc. The unsymmetric ester **14** was obtained as brown oil with 20% yield (3.6 mg, 0.009 mmol), and the acid **16** with a 45% yield (9.2 mg, 0.019 mmol).

**14**: R_f_ (PE:EtOAc/4:1): 0.646. ^1^H-NMR (400 MHz, CDCl_3_): *δ* (ppm) = 7.31 (dd, *J* = 10.8, 4.7 Hz, 5H), 7.22 (d, *J* = 7.5 Hz, 4H), 7.20–7.14 (m, 2H), 5.92 (ddt, *J* = 15.9, 11.0, 5.6 Hz, 1H), 5.16 (dd, *J* = 11.0, 1.4 Hz, 3H), 5.14–5.09 (m, 2H), 4.22 (dd, *J* = 4.2, 1.4 Hz, 4H). ^13^C-NMR (100 MHz, CDCl_3_): *δ* (ppm) = 178.4, 134.4, 134.4, 128.7, 128.7, 125.8, 120.2, 120.2, 116.5, 116.5, 116.5. HRMS [ESI] *m*/*z* calculated for C_24_H_22_O_6_ [M + Na]^+^ = 429.1314, found: 429.1311.

**16**: R_f_ (PE:EtOAc/4:1): 0.132. ^1^H-NMR (400 MHz, CDCl_3_): *δ* (ppm) = 7.37–7.26 (m, 10H), 7.26–7.09 (m, 4H), 7.06 (d, *J* = 8.2 Hz, 1H), 6.97 (d, *J* = 8.2 Hz, 1H), 6.28 (t, *J* = 5.4 Hz, 1H; OH), 5.14 (s, 2H), 5.10 (d, *J* = 4.2 Hz, 2H), 3.70 (s, 3H), 3.67–3.59 (m, 4H).^13^C-NMR (100 MHz, CDCl_3_): *δ* (ppm) = 172.5, 172.1, 154.1, 153.0, 133.6, 132.2, 130.3, 130.2, 128.8, 128.7, 128.7, 128.3, 128.3, 127.5, 117.9, 114.5, 72.1, 72.0, 66.9, 66.7, 52.3, 52.2, 40.5, 40.0. HRMS (ESI) *m*/*z* calculated for C_31_H_28_O_6_ [M + Na]^+^ = 519.1784, found: 519.1782 and calculated for C_31_H_28_O_6_ [M + H]^−^ = 495.1808, found: 495.1810.

### 4.11. 2-(2′,6-Dimethoxy-5′-(2-methoxy-2-oxoethyl)-[1,1′-biphenyl]-3-yl)acetic acid (***5***)

A total of 29.7 mg of PFL was dissolved in phosphate buffer (3.36 mL, 50 mM, pH 8.0) and **3** (15 mg, 0.042 mmol, 1.0 eq) in DMSO (2.23 mL) was added. The solution was stirred for 48 h at 37 °C. To terminate the reaction 1 M HCl was added to receive pH 1 and the aqueous layer was washed three times with EtOAc. The solvent was evaporated, and the residue was purified with preparative LC–MS as mentioned above. Compound **5** was obtained as a colorless oil with 41% yield (5.9 mg, 0.017 mmol).

^1^H-NMR (400 MHz, CDCl_3_): *δ* (ppm) = 7.26–22 (m, 2H), 7.15 (dd, *J* = 4.1, 2.3 Hz, 2H), 6.92 (dd, *J* = 8.4, 4.1 Hz, 2H), 3.75 (d, *J* = 3.6 Hz, 6H), 3.69 (s, 3H), 3.62 (s, 2H), 3.59 (s, 2H).^13^C-NMR (100 MHz, CDCl_3_): *δ* (ppm) = 176.6, 172.4, 156.3, 156.1, 132.5, 132.3, 129.5, 129.4, 127.6, 127.3, 125.7, 125.0, 111.2, 111.2, 55.8, 55.8, 52.0, 40.2, 40.0. HRMS (ESI) *m*/*z* calculated for C_19_H_20_O_6_ [M + Na]^+^ = 367.1158, found: 367.1161.

### 4.12. Benzyl 2-(2′,6-dimethoxy-5′-(2-methoxy-2-oxoethyl)-[1,1′-biphenyl]-3-yl)acetate (***15***)

Firstly, **5** (4 mg, 0.012 mmol, 1 eq) was dissolved in CH_3_CN (3 mL). DBU (0.5 mg, 0.003 mmol, 0.2 eq) and benzyl bromide (4 µL, 0.024 mmol, 2.0 eq) were added. The reaction mixture was stirred at 84 °C for 2 h. After cooling down, H_2_O was added, and the aqueous layer was washed three times with EtOAc. The combined organic layers were dried over Na_2_SO_4_ and the solvent was evaporated. The residue was purified using silica gel chromatography using a PE:EtOAc gradient (start: 100% PE, 2CV; 50%:50% PE:EtOAc, 2 CV; 100% EtOAc, **15** eluted at PE:EtOAc 50%:50%). Compound **15** was obtained as a colorless oil with 81% yield (4.1 mg, 0.009 mmol).

^1^H-NMR (400 MHz, CDCl_3_): *δ* (ppm) = 7.36–7.29 (m, 5H), 7.26–7.22 (m, 2H), 7.14 (dd, *J* = 13.1, 2.3 Hz, 2H), 6.91 (d, *J* = 8.4 Hz, 2H), 5.13 (s, 2H), 3.76 (s, 3H), 3.74 (s, 3H), 3.69 (s, 3H), 3.63 (s, 2H), 3.59 (s, 2H).^13^C-NMR (100 MHz, CDCl_3_): *δ* (ppm) = 172.34, 171.72, 162.43, 162.01, 158.74, 156.20, 156.17, 132.37, 132.31, 129.44, 129.38, 128.50, 128.14, 128.09, 127.58, 127.57, 125.65, 125.58, 111.67, 111.16, 77.32, 77.00, 76.68, 66.52, 55.80, 55.78, 51.99, 40.47, 40.32. HRMS (ESI) *m*/*z* calculated for C_26_H_26_O_6_ [M + Na]^+^ = 457.1627, found: 457.1624.

### 4.13. Molecular Docking

All compounds were sketched in Schrödinger Maestro (Schrödinger Release 2019-3: Maestro; Protein Preparation Wizard; Epik; Impact, Schrödinger, LLC, New York, NY, USA, 2019), and energy minimized with Schrödinger MacroModel (Schrödinger Release 2019-3: MacroModel, Schrödinger, LLC, New York, NY, USA, 2019) and the OPLS3 force field [40] to a gradient of 10^-4^ kJ·mol^−1^Å^−1^. Molecular docking was performed using Autodock 4.2 (MGL Tools) [41] and the Lamarckian genetic algorithm [42]. Protein coordinates were obtained from the pdb database (PDB codes: CRL: 1TRH [43], MML: 4TGL [44], RNL: 1LGY [45], PCL: 3LIP [46]) and preprocessed with the Protein Preparation Wizard of the Schrödinger Suite and AutodockTools. The search space covered the catalytic center of the proteins. 

## Figures and Tables

**Table 1:** Results of the colorimetric activity assay of nine lipases and their activity to hydrolyze the ester groups in compounds **13**, **2**, and **3**. +: color change→hydrolysis, –: no color change→no hydrolysis.

Lipase	TAG 13	Biphenyl Ester 2	Biphenyl Ester 3
ANL	+	–	–
CALB	+	+	+
CRL	+	–	–
MML	+	–	–
ROL	+	–	–
RNL	+	–	–
PPL	+	–	–
PCL	+	–	–
PFL	+	–	–

Lipases: *Aspergillus niger* Lipase (ANL), *Candida antarctica* Lipase B (CALB), *Candida rugosa* Lipase (CRL), *Mucor miehei* Lipase (MML), *Rhizopus oryzae* Lipase (ROL), *Rhizopus niveus* (RNL), Porcine pancreas Lipase (PPL), *Pseudomonas cepacia* Lipase (PCL), *Pseudomonas fluorescens* Lipase (PFL).

**Table 2:** In silico predicted free binding energies of biphenyl ester **3** to lipases CRL, RNL, MML, and PFL. *Δ*G^0^ values were computed with AutodockTools.

Predicted Affinity of 3 to Lipase	CRL	RNL	MML	PFL
*Δ*G^0^ (kcal · mol^−1^)	−3.23	−5.64	−6.66	−6.79

## Supplementary Materials

The following supplementary materials are available online. Scheme S1: Scheme illustrating the mechanism of the biomimetic Fe(III)-catalyzed oxidative phenolic coupling for the synthesis of biphenyl esters. Fe(III) is reduced by electron acceptance of aromatic electrons. Radicals and charges can be stabilized by mesomeric effects of the electron donating ortho substitution. Scheme S2: Formation of byproduct 16 during the two-step protocol. Byproduct **16** is formed at 40% yield showing that the acid group is less reactive than phenolic hydroxyl groups when intermediate **4** is not isolated. Figure S1: Colorimetric lipase assay of nine lipases from different organisms. A color change from blue to yellow indicates the conversion of ester groups to free carboxylic acids. The hydrolysis activity of the lipases is markedly dependent on the organic solvent, since no catalytic activity of any lipase was detectable with acetonitrile (CH_3_CN) or toluene. Figure S2: UV signals and ratios of compounds **2** (black), **4** (blue), and **6** (green), obtained by analytical LC–MS reveal the pH-dependency of the chemoselective biphenyl ester hydrolysis. Figure S3: UV signals and ratios of compounds **3** (black), **5** (blue), and **7** (green) obtained by analytical LC–MS show that CALB converses compound **3** directly to **7**. Figure S4: UV signals and ratios of compounds **10** (black), **11** (green), and **12** (blue) obtained by analytical LC–MS. Table S1: Ratios of biphenyl ester **3** to asymmetric acid **5** after different reaction times and at different temperatures and conversion with lipases CRL, MML, RNL, and PFL (40% DMSO (*v*/*v*)). Figures S5–S7: Compound characterization data (UV absorption and mass spectra of relevant compounds).
