# Supplementary material for: Lipase-Catalyzed Chemoselective Ester Hydrolysis of Biomimetically Coupled Aryls for the Synthesis of Unsymmetric Biphenyl Esters
Source: Molecules. 2019 Nov 23;24(23):4272. doi: 10.3390/molecules24234272 (PMC6930668; doi:10.3390/molecules24234272)
Supplement: Supplementary file 1 [file molecules-24-04272-s001.pdf]

Supplementary Materials

Lipase-Catalyzed Chemoselective Ester Hydrolysis of Biomimetically Coupled Aryls for the Synthesis of Unsymmetric Biphenyl Esters

Janna Ehlert, Jenny Kronemann, Nadine Zumbrägel and Matthias Preller*

[1] Institute for Biophysical Chemistry, Hannover Medical School, 30625 Hannover, Germany
* Correspondence: preller.matthias@mh-hannover.de

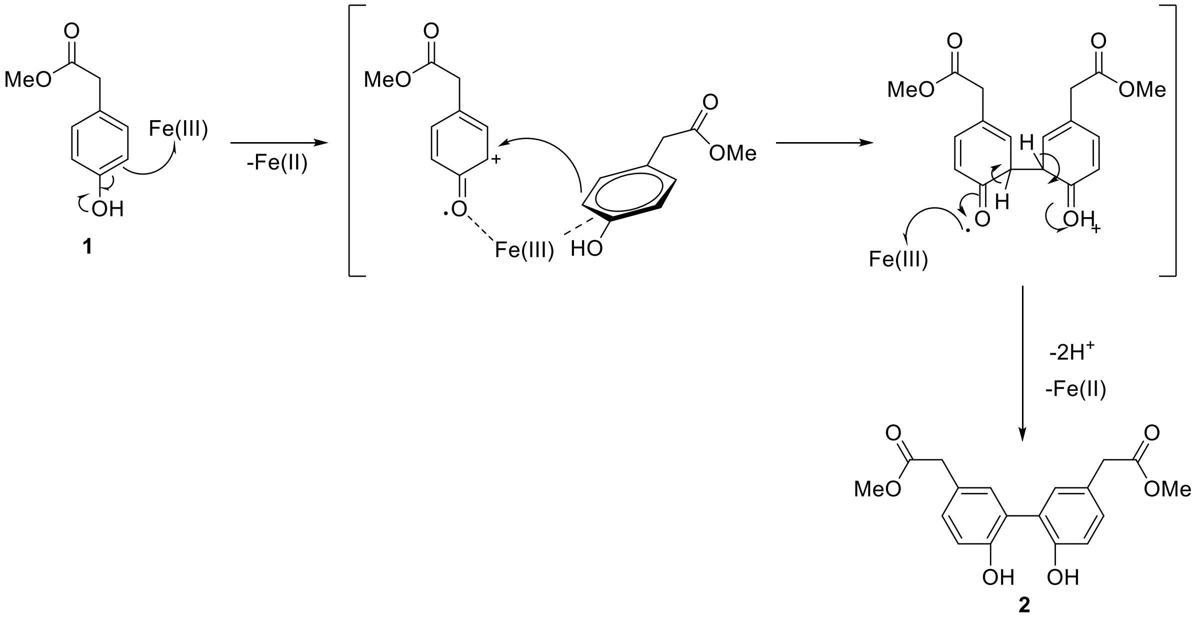

Scheme S1: Scheme illustrating the mechanism of the biomimetic Fe(III)-catalyzed oxidative phenolic coupling for the synthesis of biphenyl esters. Fe(III) is reduced by electron acceptance of aromatic electrons. Radicals and charges can be stabilized by mesomeric effects of the electron donating *ortho* substitution.

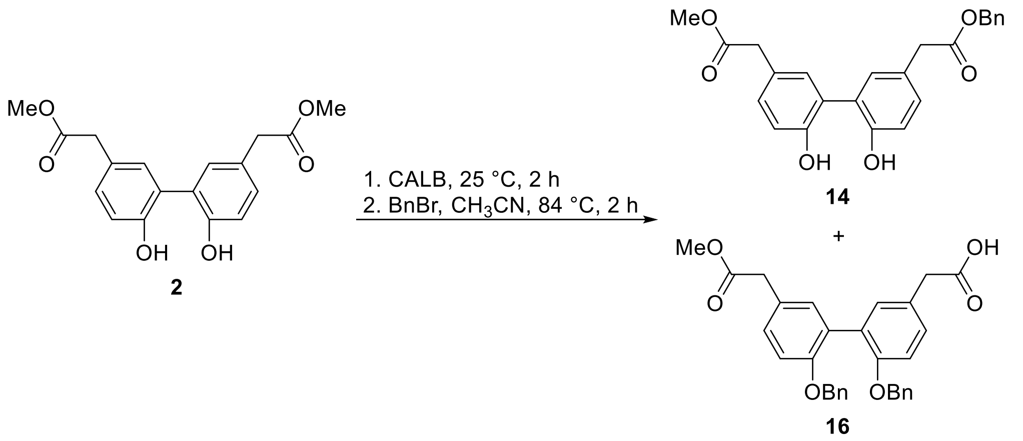

Scheme S2: Formation of byproduct 16 during the two-step protocol. 16 is formed at 40% yield, showing that the phenolic hydroxyl groups can react with BnBr, instead of the acid group when intermediate **4** is not isolated.

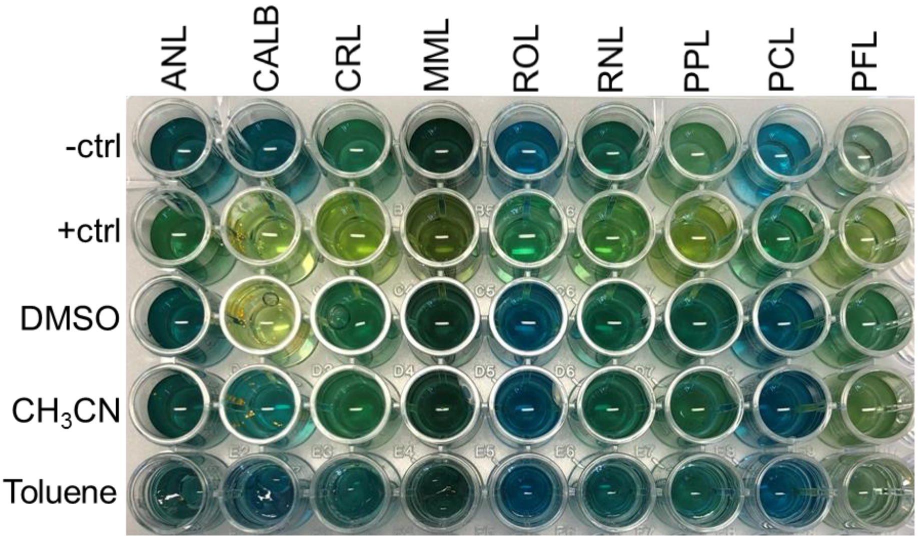

Figure S1: Colorimetric lipase assay of nine lipases from different organisms. A color change from blue to yellow indicates the conversion of ester groups to free carboxylic acids. The hydrolysis activity of the lipases is markedly dependent on the organic solvent, since no catalytic activity of any lipase was detectable with acetonitrile (CH_3CN) or toluene.

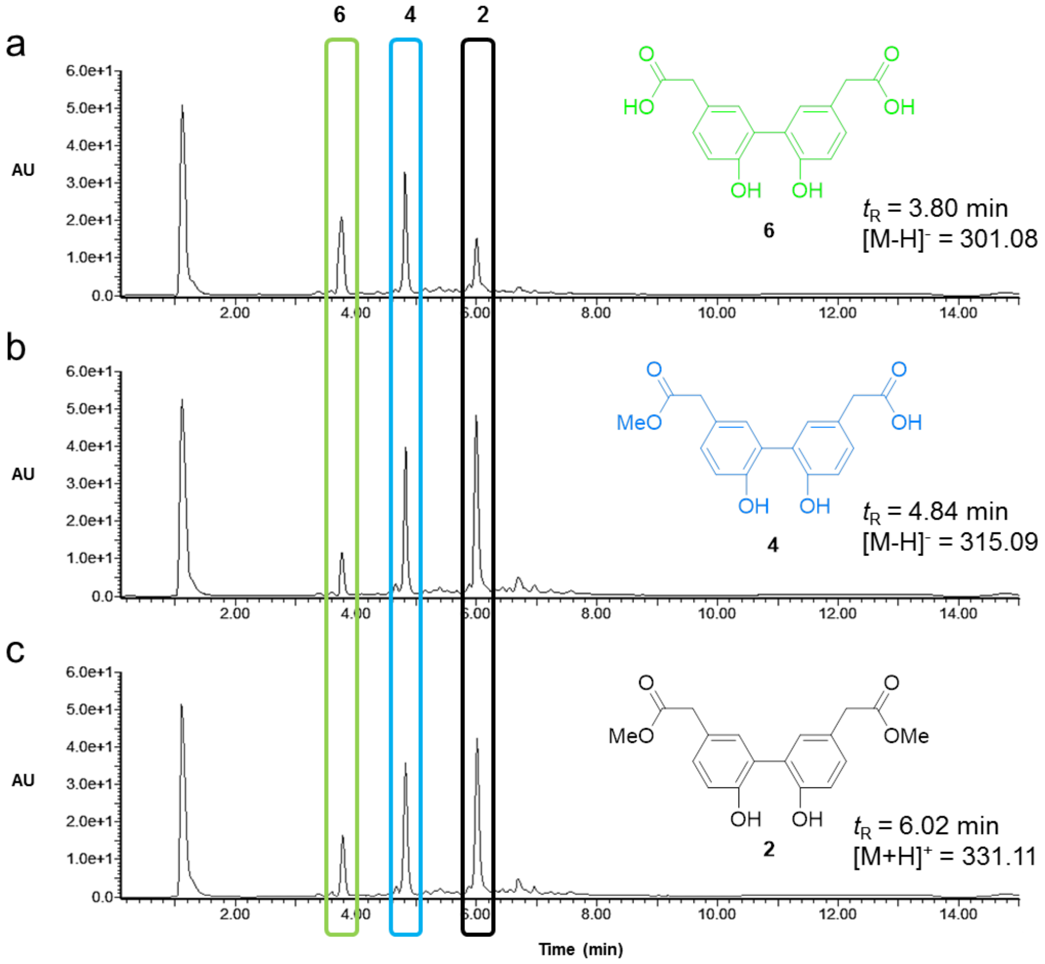

Figure S2: pH dependency of the chemoselective hydrolysis of biphenyl ester 2 with free hydroxyl groups at the core moiety obtained by analytical LCMS and comparison of the UV-signal ratios of biphenyl esters 2 (black), 4 (blue) and 6 (green). All reactions were performed at 25 °C and

10% DMSO (v/v) for 4 h using CALB. a) pH 9.0; b) pH 8.0; c) pH 7.4. The chemical structures, retention times and m/z values for compounds **2**, **4**, and **6** are given.

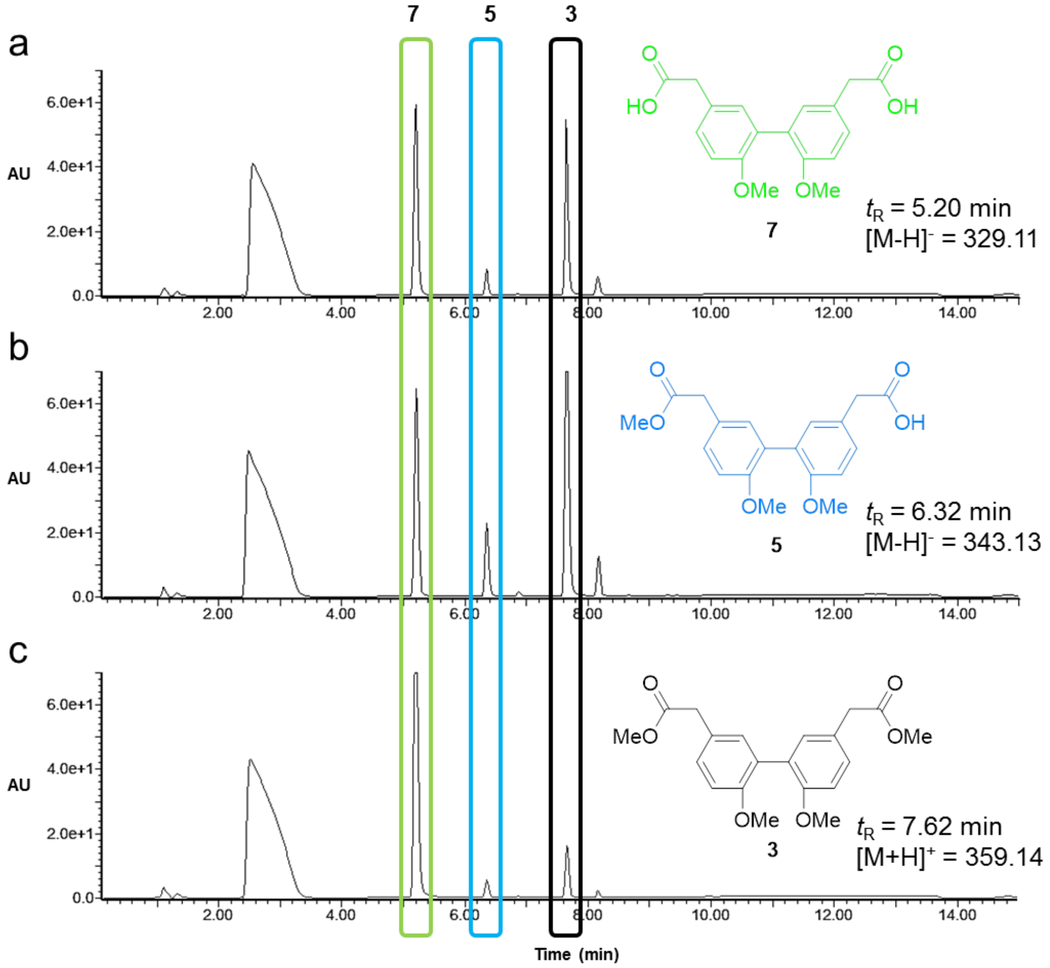

Figure S3: pH dependency of the chemoselective hydrolysis of biphenyl ester 3 with methoxy groups at the core moiety obtained by analytical LCMS and comparison of the UV-signal ratios of biphenyl esters 3 (black), 5 (blue) and 7 (green). All reactions were performed at 25 °C and 10% DMSO (v/v) for 90 min using CALB. a) pH 9.0; b) pH 8.0; c) pH 7.4. The chemical structures, retention times and m/z values for compounds **3**, **5**, and **7** are given

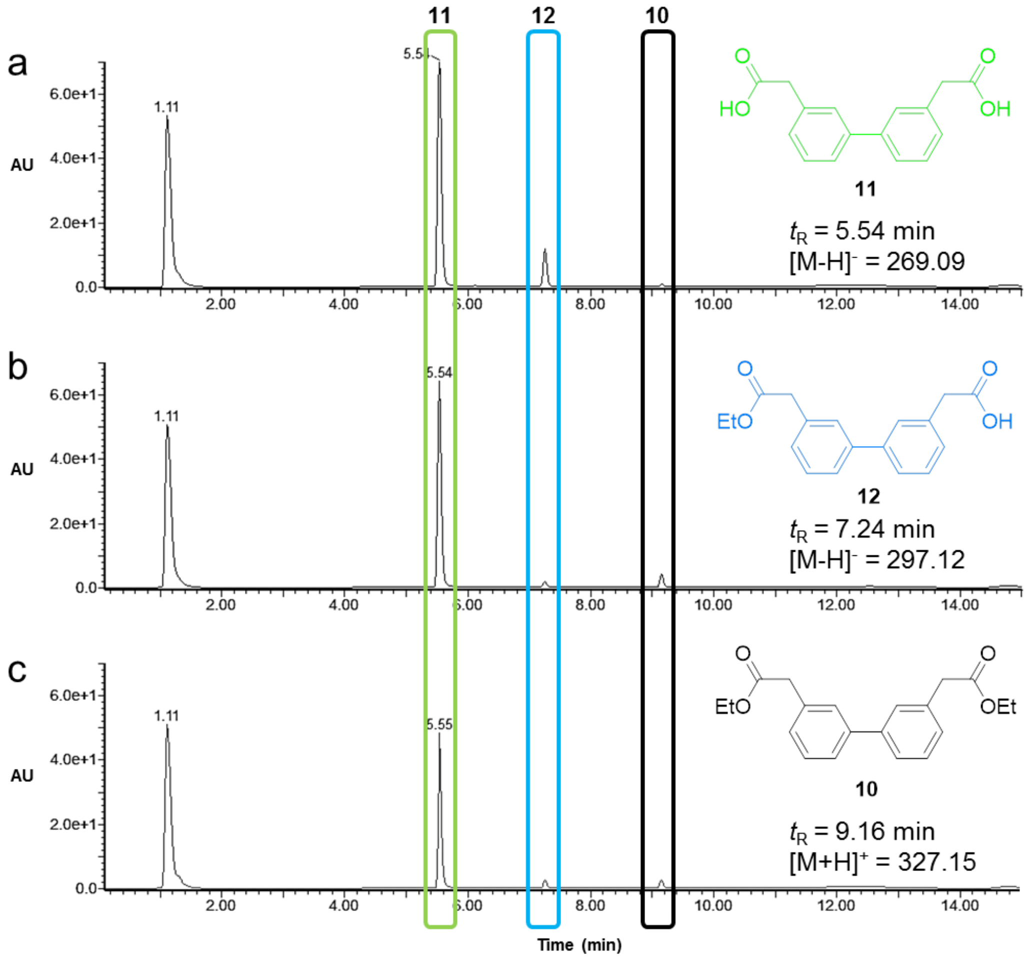

Figure S4: pH dependency of the hydrolysis of biphenyl ester 10 without *ortho* substituents at the core moiety obtained by analytical LCMS and comparison of the UV-signal ratios of biphenyl esters 10 (black), 11 (green) and 12 (blue). All reactions were performed at 25 °C and 10% DMSO (v/v) for 90 min using CALB. a) pH 9.0; b) pH 8.0; c) pH 7.4. The chemical structures, retention times and m/z values for compounds **2**, **4**, and **6** are given. Note the fast conversion to the symmetric acid **11**.

Table S1: Obtained reactant (**3**):unsymmetric biphenyl ester (**5**) ratios as determined from analytical LCMS at systematically varied reaction times and temperatures for lipases CRL, MML, RNL and PFL in the presence of 40% DMSO (v/v).

**Lipase**	**Reaction time (h)**	**25 °C**		**30 °C**		**37 °C**	
		**3**	**5**	**3**	**5**	**3**	**5**
CRL	2	34	1	40	1		
MML		16	1	18	1	10.3	1
RNL		28	1	36	1		
PFL		10	1	20	1	14	1
CRL	4	40	1	nd		nd	
MML		18	1				
RNL		36	1				
PFL		10	1				
MML	5	nd		nd		10	1
PFL						10.4	1
CRL	17	nd		30	1	nd	

MML				18	1		
RNL				36	1		
PFL				17	1		
MML	22	nd		nd		7	1
PFL						6.25	1
CRL		20	1				
MML	24	15	1	nd		nd	
RNL		20	1				
PFL		10	1				
CRL						6.7	1
MML	48	nd		nd		3.6	1
RNL						6	1
PFL						3.5	1

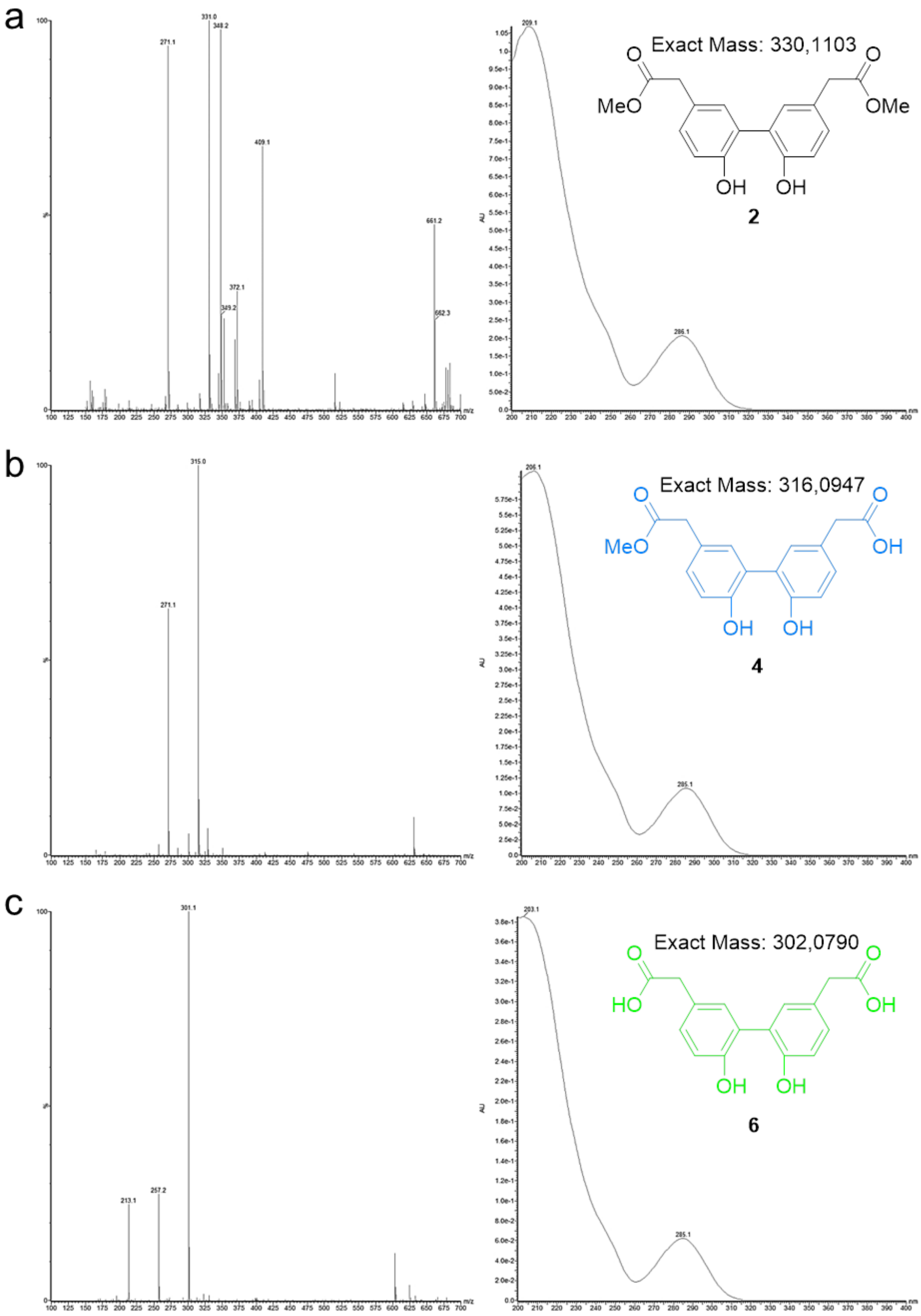

Figure S5: Mass spectra and UV absorption spectra of compounds 2 (a) 4 (b) and 6 (c).

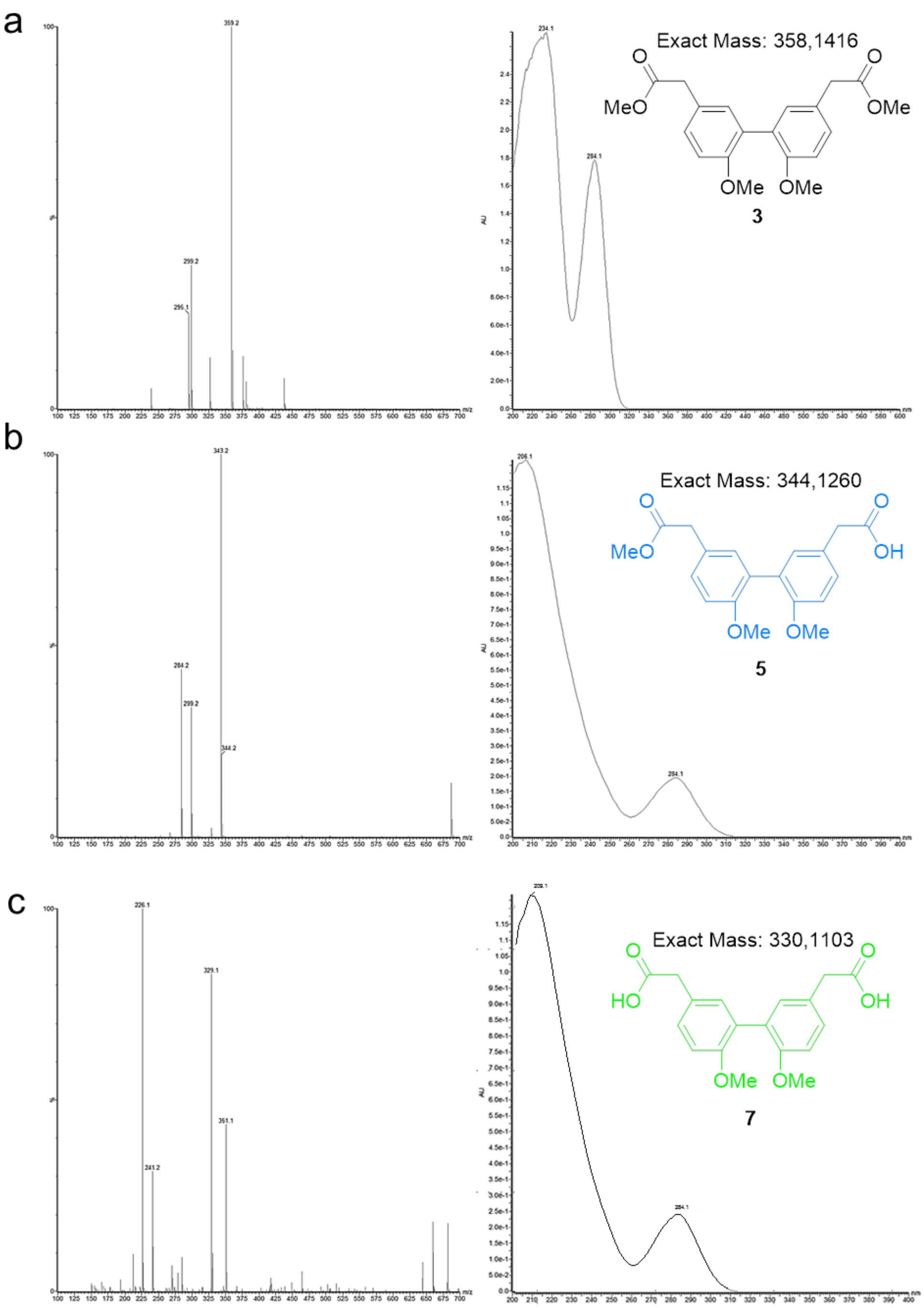

Figure S6: Mass spectra and UV absorption spectra of compounds 3 (a), 5 (b), and 7 (c).

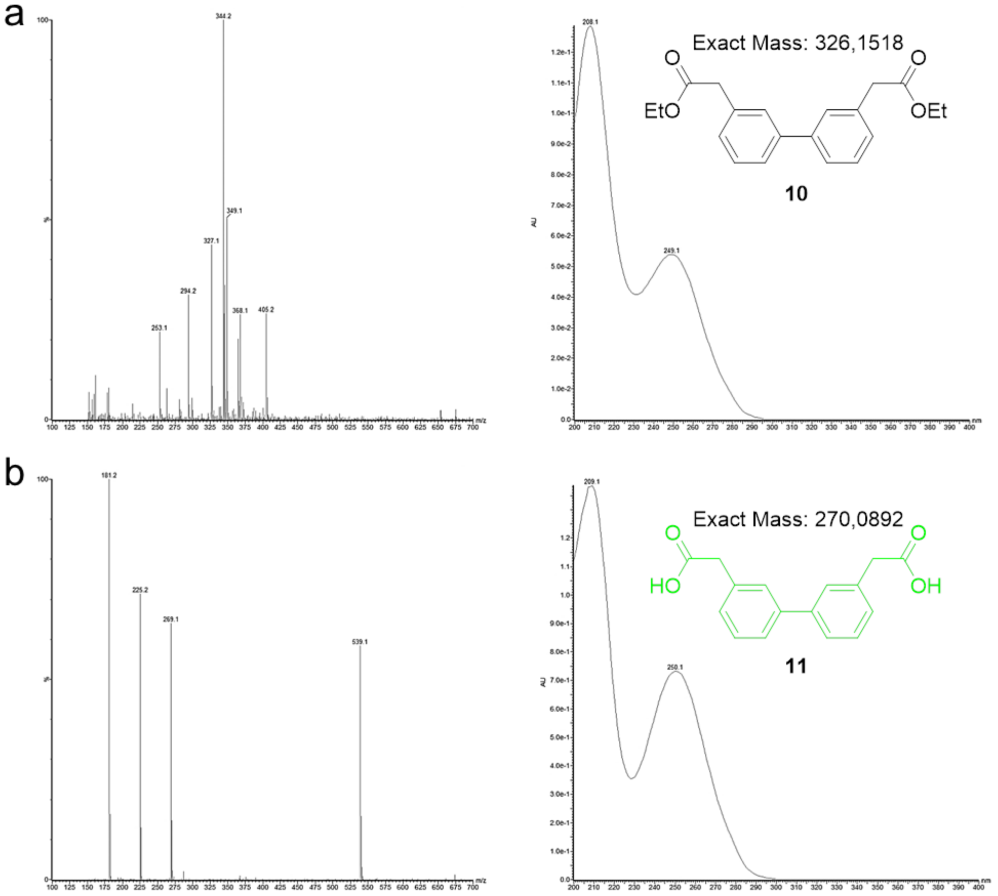

Figure S7: Mass spectra and UV absorption spectra of compounds 10 (a) and 11 (b).
